# Generic Insect Repellent Detector from the Fruit Fly *Drosophila melanogaster*

**DOI:** 10.1371/journal.pone.0017705

**Published:** 2011-03-16

**Authors:** Zainulabeuddin Syed, Julien Pelletier, Eric Flounders, Rodrigo F. Chitolina, Walter S. Leal

**Affiliations:** Department of Entomology, University of California Davis, Davis, California, United States of America; Center for Genomic Regulation, Spain

## Abstract

**Background:**

Insect repellents are prophylactic tools against a number of vector-borne diseases. There is growing demand for repellents outperforming DEET in cost and safety, but with the current technologies R&D of a new product takes almost 10 years, with a prohibitive cost of $30 million dollar in part due to the demand for large-scale synthesis of thousands of test compounds of which only 1 may reach the market. R&D could be expedited and cost dramatically reduced with a molecular/physiological target to streamline putative repellents for final efficacy and toxicological tests.

**Methodology:**

Using olfactory-based choice assay we show here that the fruit fly is repelled by not only DEET, but also IR3535 and picaridin thus suggesting they might have “generic repellent detector(s),” which may be of practical applications in new repellent screenings. We performed single unit recordings from all olfactory sensilla in the antennae and maxillary palps. Although the ab3A neuron in the wild type flies responded to picaridin, it was unresponsive to DEET and IR3535. By contrast, a neuron housed in the palp basiconic sensilla pb1 responded to DEET, IR3535, and picaridin, with apparent sensitivity higher than that of the DEET detectors in the mosquitoes *Culex quinquefasciatus* and *Aedes aegypti*. DmOr42a was transplanted from pb1 to the “empty neuron” and showed to be sensitive to the three insect repellents.

**Conclusions:**

For the first time we have demonstrated that the fruit fly avoids not only DEET but also IR3535 and picaridin, and identified an olfactory receptor neuron (ORN), which is sensitive to these three major insect repellents. We have also identified the insect repellent-sensitive receptor, DmOr42a. This generic detector fulfils the requirements for a simplified bioassay for early screening of test insect repellents.

## Introduction

Arthropod-borne diseases cause considerable human suffering and death. Mosquitoes, in particular, are notorious for their deleterious transmission of pathogens and parasites while feeding on human blood. *Anopheles* mosquitoes, particularly *An. gambiae* and *An. funestus*, are implicated in the deaths of about one million humans, particularly women and children, every year [1]. While feeding on their victim's blood, they unwittingly transmit the malaria-causing parasite that threatens half of the world's population. Globally, the number of people who get malaria each year is greater than the population of the United States [2]. The yellow fever mosquito, *Aedes aegypti*, is the primary vector of dengue throughout the tropical and subtropical world, thus accounting every year for several million cases globally [3]. *Culex* mosquitoes are major vectors of pathogens including the human filarial nematode, *Wuchereria bancrofti*, and arboviruses such as St. Louis encephalitis, Japanese encephalitis, Venezuela equine encephalitis, Western equine encephalitis and West Nile virus [4]. Newborn babies and immunocompromised patients from endemic areas, as well as military personnel and travelers moving into these areas, are at particularly higher risk given that typically they do not have immunity to pathogens locally transmitted by mosquitoes. Insect repellents are prophylactic tools against all these maladies. They may be used in conjunction with bednets and other integrated vector management (IVM) tools to reduce mosquito bites [5], [6], [7], [8], but typically they are applied to the skin of uncovered parts of the body.

Despite its safety record [9], there is growing concern regarding topical applications of DEET (IUPAC name: *N,N*-diethyl-3-methylbenzamide) at high concentrations because deeper skin penetration can cause potential toxicity [10]. Additionally, DEET does not fulfill the ideal properties of insect repellents [9]. For example, DEET is a plasticizer that reacts with synthetic rubber and certain plastics and has several cosmetic concerns, including unpleasant odor. More importantly, most DEET formulations have a short duration of action (limited to seven hours) [10], which is a serious hindrance for military use as well as for civilians residing in areas with high temperatures. However, since its discovery more than five decades ago [11], DEET remains the most effective repellent in use today [12], and only a handful of new products have reached the market in the United States, particularly IR3535 (IUPAC name: 3-(*N*-acetyl-*N*-butyl)aminopropionic acid ethyl ester) and picaridin (IUPAC name: (*RS*)-sec-butyl 2-(2-hydroxyethyl)piperidine-1-carboxylate; also known as icaridin, KBR3023, and Bayrepel). With the current technology, it takes about 10 years and approximately $30 million to develop a new repellent [10] because only 1 out of 20,000 compounds reach the market [10]. Typically, insect repellents have broad spectrum activity against not only blood-feeding arthropods of medical importance (e.g.: mosquitoes, sand flies, ticks), but also insect in general (e.g.: cockroaches [13] and the fruit fly [14]). The cost of producing novel repellents becomes prohibitive in part because conventional evaluations [15] against a number of arthropods of medical importance require large-scale synthesis of thousands of compounds in the early stages of research and development (R&D). This could be alleviated by (*i*) replacing trial-and-error approaches with molecular/physiological target-based simple bioassays to screen test compounds at the early stages of development, and (*ii*) limiting large-scale synthesis for conventional evaluations of only biochemically/physiologically active compounds. Hitherto, progress has been retarded because of the lack of molecular and/or physiological targets for these “reverse chemical ecology” approaches. We suggest that this lacuna can now be filled with the fruit fly, which as shown here is endowed with sensilla housing a “generic repellent detector,” i.e., an olfactory receptor neuron (ORN) expressing an odorant receptor (OR) sensitive to DEET, IR3535, and picaridin. This system would not require large-scale chemical synthesis as minute amounts of test compounds suffice for preliminary screenings.

## Results and Discussion

### Flies are repelled by DEET, IR3535, and picaridin

**Figure 1:**
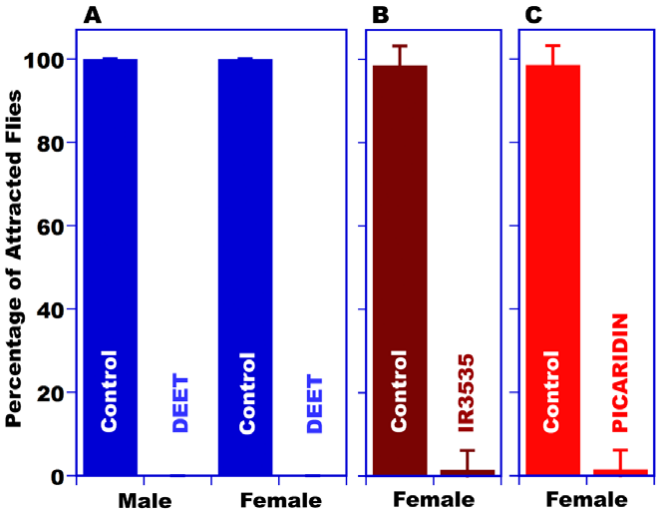
Repellency assay indicating avoidance of *D. melanogaster* to three insect repellents: DEET, IR3535, and picaridin. (*A*) Male and female flies responded equally to DEET (*N* = 180 flies tested). Female flies avoid entering the food chambers treated with (B) IR3535 (*N* = 90 flies tested) and (C) picaridin (*N* = 90 flies tested). Data are from 9 independent trials for each test, with ten flies used in each trial.

Using a previously described choice assay [14], we showed that the fruit fly is indeed repelled by DEET [14], [16], with no difference between male and female responses (Fig. 1A). Flies placed in Petri dishes having food available only inside two food chambers (1.5 ml micro centrifuge, “Eppendorf like” tubes) crossed control filter paper strips (solvent only) placed at the entrance of these chambers, reached out to the food source, and remained trapped inside the feeding chambers (*N* = 180 flies, 100%). By contrast, in no occasion (*N* = 18 trials, 10 flies per trial) have we observed flies entering chambers treated with DEET (Figs. 1A). Under similar conditions, flies were also repelled by IR3535 and picaridin (Fig. 1B,C), and in each case only 1 out of 90 entered the treated chambers. The paradigm of the choice assay we used [14] suggests that the observed repellency to DEET, IR3535, and picaridin (Fig. 1) is mediated by the fly's olfactory system. We then reasoned the olfactory system of the fruit fly houses ORN(s) sensitive to these insect repellents, which - as previously suggested [17] - might be detected through non-contact chemosensation.

### Scanning the fruit fly antennae for generic repellent detector(s)

**Figure 2:**
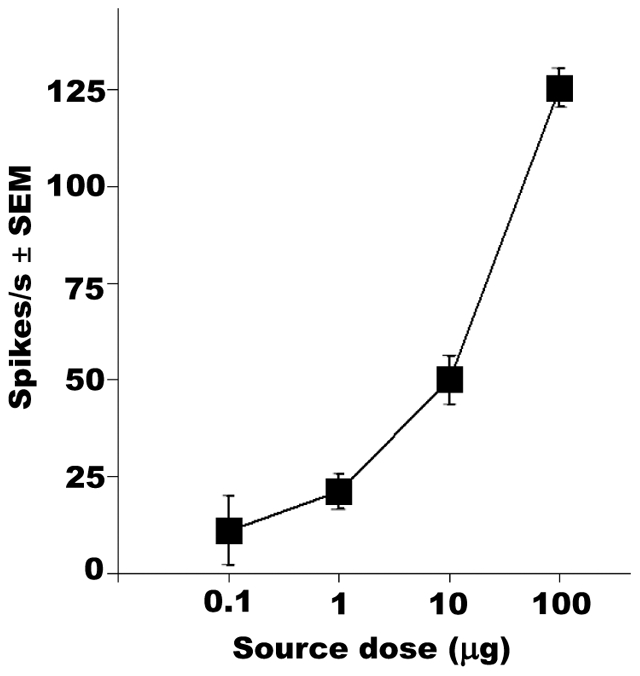
Dose-dependent excitatory responses from a picaridin-sensitive ORN housed in an antennal basiconic sensillum ab3 on *D. melanogaster* antennae. Hexane (control) responses were subtracted. (*N* = 7). Error bars are standard error of the mean (SEM).

**Figure 3:**
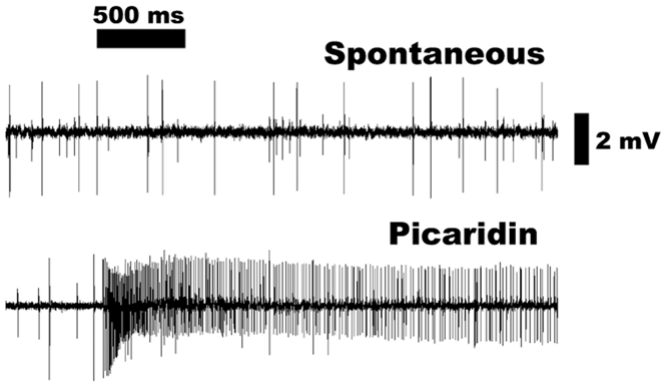
Extracellularly recorded single unit responses from an ab3 sensillum. Spontaneous activity (upper trace) and picaridin-induced excitatory response (lower trace) from the large amplitude neuron, ab3A. Source dose, 100 µg. Note the excitatory responses lasted beyond the stimulus period.

We scanned all olfactory sensilla in the antennae of the fruit fly by single sensillum recordings using DEET, IR3535 and picaridin as stimuli. During this mapping, at least three sensilla of each type (basiconic, coeloconic, and trichoid) were challenged with these insect repellents. Although we did not find a single ORN sensitive to DEET or IR3535, one neuron housed in ab3 sensilla responded to picaridin with high sensitivity (threshold 0.1 µg, source dose) (Fig. 2). Based on the large spike amplitude (Fig. 3), the picaridin-sensitive neuron was identified as ab3A, which is known to harbor DmOr22a/b [18]. Interestingly, signal termination of picaridin was very slow (Fig. 3). Normally spike frequencies decrease immediately at the end of the stimulus (see below) [19]. Considering this unusual signal termination and, more importantly, due to its insensitivity to two other insect repellents, ab3A neuron is not a good candidate for screening new insect repellents.

### ORN in the maxillary palps is sensitive to DEET and other repellents

**Figure 4:**
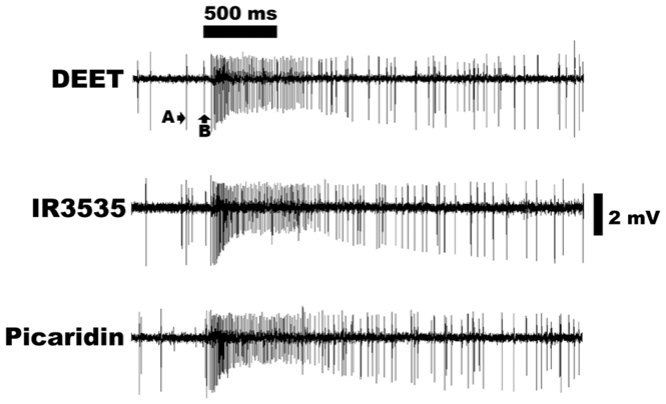
Excitatory responses from an ORN housed in the maxillary palp basiconic sensillum pb1A when challenged with three insect repellents: DEET, IR3535 and picaridin. Two types of neurons, A and B, are identified on the basis of their spike amplitudes. The ORN with larger amplitude, A, is stimulated by the three insect repellents, whereas the neuron with smaller amplitude, B, was unresponsive. Source dose, 100 µg.

**Figure 5:**
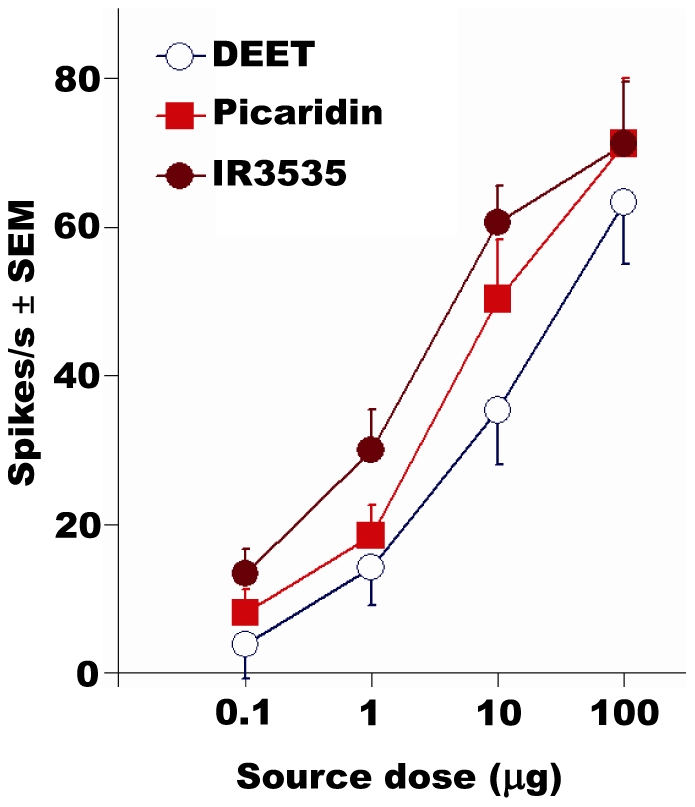
All three insect repellents induce dose-dependent excitatory responses in the pb1A ORN. Hexane (control) responses were subtracted. (*N* = 7). Error bars are standard error of the mean (SEM).

**Figure 6:**
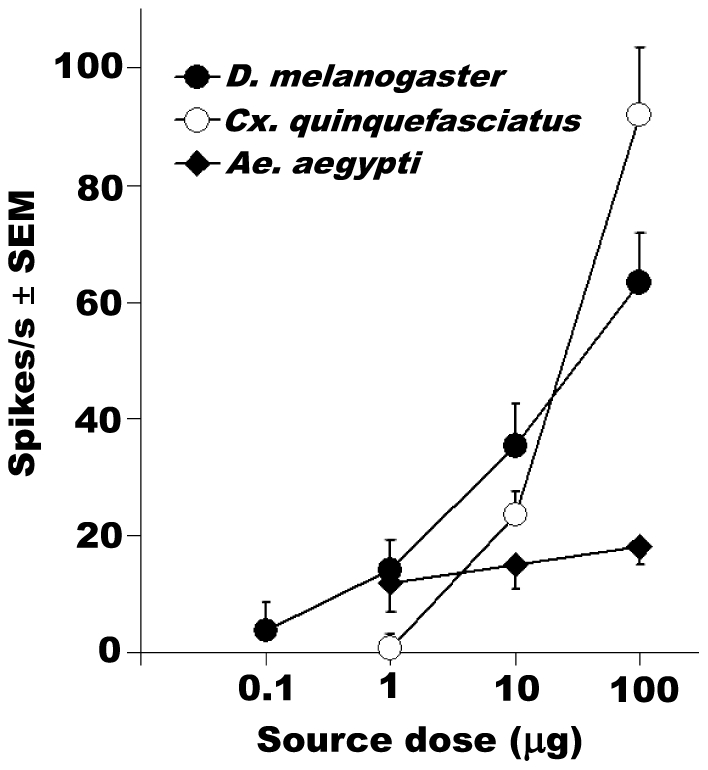
Comparative DEET-elicited responses. DEET-induced excitatory responses from *Drosophila* pbA1 ORN showed lower threshold than those recorded from the DEET-sensitive mosquito ORNs from the Southern House mosquito, *Cx. quinquefasciatus* [20] and the yellow fever mosquito, *Aedes aegypti* [21], respectively. Error bars are standard error of the mean (SEM).

**Figure 7:**
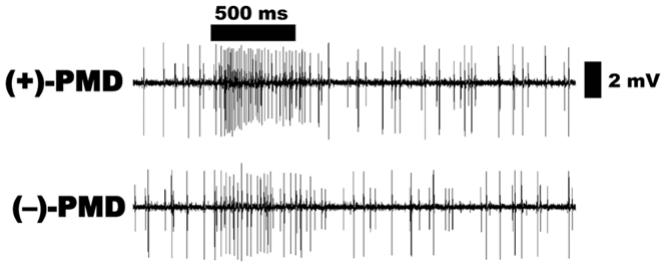
Repellent-sensitive ORN, pb1A, challenged with PMD stereoisomers. (+)-PMD elicited higher response from the large spike neuron than (−)-PMD. Source dose, 100 µg.

Next, we performed single unit recordings from all olfactory sensilla in the maxillary palps and found an ORN in the basiconic sensilla pb1 that responded to DEET (Fig. 4) in a dose-dependent fashion (Fig. 5). Surprisingly, these sensilla showed apparent higher sensitivity (lower threshold) to DEET than sensilla previously identified in the Southern house [20] and the yellow fever [21] mosquitoes (Fig. 6). In contrast to mosquito ORNs, the DEET-detecting neuron in the fruit fly is a “generic repellent detector.” In addition to DEET it responded dose-dependently to IR3535 and picaridin (Fig. 4,5). Interestingly, this repellent-detecting ORN discriminates enantiomers of PMD (IUPAC name: 2-(1-hydroxy-1-methylethyl)-5-methylcyclohexanol), a repellent derived from natural sources (Fig. 7). This is particularly interesting given that behavioral assays showed that a stereoisomer of another insect repellent, (1*S*,2*S*)-2-methylpiperidinyl-3-cyclohexene-1-carboxamide, is 2.5 times as effective as the racemic mixture against *Aedes aegypti* [12].

The two ORNs housed in the pb1 sensilla of the fruit fly were clearly distinguished (Fig. 4) by their odorant response profiles and the amplitude of their spikes [22], [23]. The neurons with larger and smaller spike amplitudes are named ORN-A and ORN-B, respectively [22], [23] (Fig. 4). In agreement with previous studies [22], [23], ORN-A responded to ethyl acetate, ethyl propionate, isoamyl acetate, (*E*)-2-hexenal, and heptan-2-one, but not to 4-methylphenol. By contrast, ORN-B was stimulated by 4-methylphenol, but was silent to the other odorants. Therefore, we were able to unambiguously conclude that DEET, IR3535, and picaridin stimulated ORN-A, but not ORN-B. It is technically challenging to correlate the previously discovered DEET-sensitive ORNs from the Southern house mosquito [20] with the ORs in the *Culex* genome [24], [25]; same is true for *Ae. aegypti*. However, the wealth of information on the mapping of *Drosophila* ORs vis-à-vis ORNs [18], [23], [26], [27] allows us to identify the putative insect repellent receptor in the fruit fly. It has been previously demonstrated [23], and later corroborated [26], that ORN-A of the pb1 sensilla expresses the odorant receptor DmOr42a ( = Or42a). This prompted us to test Or42a expressed in the “empty neuron system” [18].

### Response of Or42a in the “empty neuron” to insect repellents

**Figure 8:**
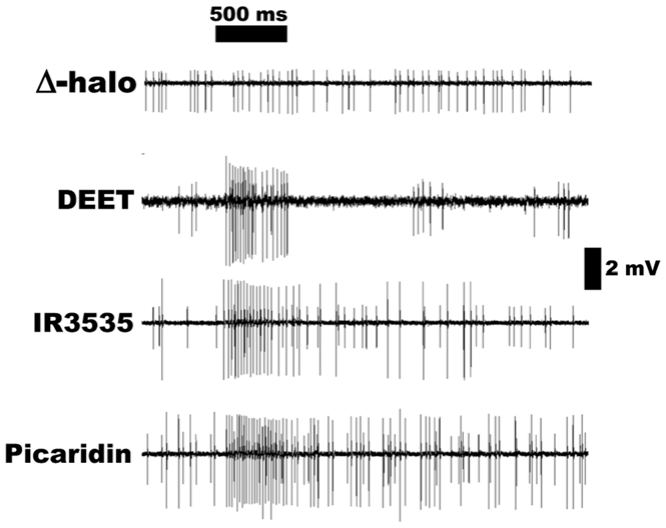
Action potentials recorded from ab3 sensilla. Δhalo flies showed spontaneous activity of neuron B, but not A, thus showing the ab3A is indeed “empty.” Lower traces were excitatory responses induced by DEET, IR3535, and picaridin and recorded from ab3 sensilla of Or42-expressing flies (w; Δhalo; UAS-DmOr42a/Or22a-GAL4).

**Figure 9:**
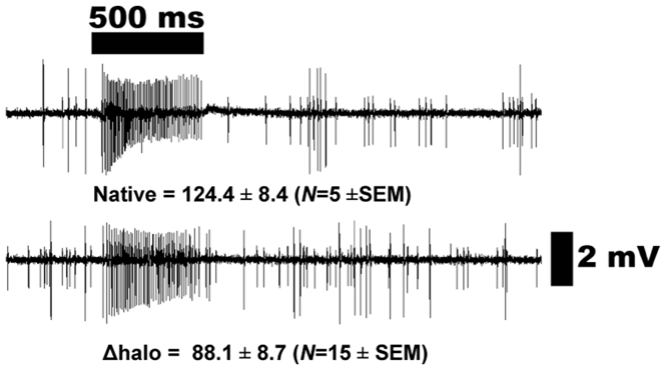
Comparative responses of Or42a expressed in its native environment and in the “empty neuron.” Ethyl butyrate (source dose, 10 µg) elicited higher responses from pb1 sensilla of wild type flies (top trace) than from the ab3 sensilla of Or42a-expressing flies (w; Δhalo; UAS-DmOr42a/Or22a-GAL4) (lower trace). SEM, standard error of the mean.

**Figure 10:**
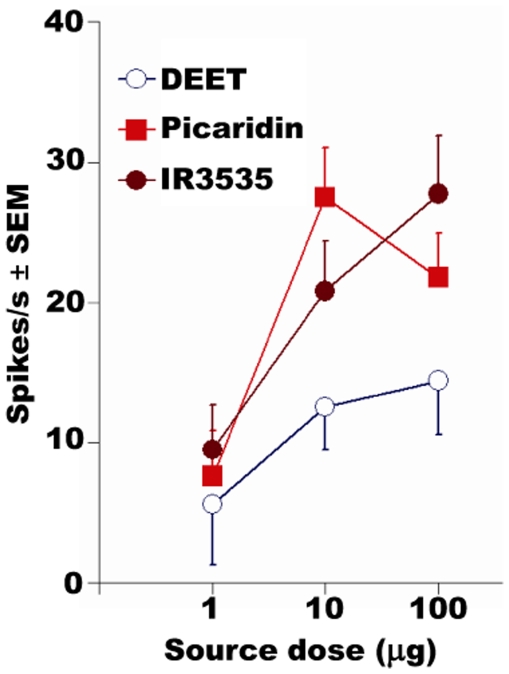
Dose-dependent responses from ab3A neuron of transgenic flies expressing Or42a (w; Δhalo; UAS-DmOr42a/Or22a-GAL4). Test flies were challenged with DEET, IR3535, and picaridin (*N* = 15). Error bars are standard error of the mean (SEM).

We performed single unit recordings from the “empty neuron” system of Or42a-expressing fruit fly (Fig. 8). Recordings from the ab3 sensilla of the Δhalo background flies showed complete absence of large amplitude spikes (spike A) when challenged with DEET, IR3535 or picaridin (Fig. 8, top trace). The transgenic flies (w; Δhalo; UAS-Or42a/Or22a-GAL4) showed spontaneous activity of ORN-A and B (large and small spikes), as expected when heterologous expression is achieved [18]. We noticed, however, that in our hands the maximal response of ab3A neuron to one of the best ligands, ethyl butyrate, was much lower (88.1±8.7 spikes/s; source dose, 10 µg) than that recorded from wild type flies (Fig. 9), as well as reported in the literature [28]. To make certain that the observed low responses were not generated by a weak driver, we performed a second crossing with newly received Or22a-Gal4 flies (a gift from Dr. J. R. Carlson). Again, Or42a expressed in the empty neuron gave 2.5x lower response to ethyl butyrate than previously observed [28]. When stimulated with DEET, IR3535, and picaridin Or42a responded, albeit with low sensitivity, in a dose-dependent fashion, except for picaridin at the highest dose tested (Fig. 10). Although the “empty neuron” has been demonstrated to be an invaluable system for testing/deorphanizing antennal ORs from the fruit fly [27] and other insects [29], it is not entirely surprising that a transplanted receptor does not perform well in the system [27], [29]. After all, odorant-binding proteins, odorant-degrading enzymes and other olfactory proteins are not transplanted along with test ORs. Low CO_2_ responses recorded from the “empty neuron” expressing the gustatory receptor Gr21a (co-expressed with Gr63a) [30], [31] have now been demonstrated to be due to the lack of the G-protein Gαq [32]. Likewise, the unavailability of other olfactory protein(s) may explain why the bombykol receptor from the silkworm moth, BmorOR1, is very sensitive to bombykol when expressed in T1 trichoid sensilla [19], but not in the “empty neuron” [33]. In the ”empty neuron” the sensitivity was enhanced by co-expression of the silkworm pheromone-binding protein BmorPBP1 [33]. It is conceivable that the absence of other olfactory protein(s) in the ab3 sensilla led to the lower responses to DEET, IR3535, and picaridin recorded from the “empty neuron system” (Fig. 9) when compared to those obtained from the endogenous ORN (Fig 5). Additionally, limited expression of Or42a, as indicated by 2.5x lower responses to ethyl butyrate, may have contributed to the weaker responses to insect repellents elicited by Or42a expressed in the “empty neuron.” It remains an interesting question for future research to determine if other olfactory protein(s) account for the differences in Or42a responses to insect repellents in endogenous and exogenous systems.

### Conclusion

Apparently, DEET has multiple modes of action. When tested at higher dosages it may act like an insecticide [34]. Recently, it has elegantly been demonstrated to be a feeding deterrent [17]. While gustatory receptors involved in this taste context [17] and an OR from larvae of the malaria mosquito have been previously identified [35], DEET odorant receptors from adult insects were hitherto *terra incognita*. The literature is even dichotomous regarding direct and indirect detection of DEET. One school favors a mode of action by “jamming” reception of other odorants [36], [37], but antennal ORNs for direct detection of DEET have been identified from both the Southern House mosquito [20] and yellow fever mosquito [38]. Although it was not possible to unambiguously correlate ORN excitation vis-à-vis behavior as repellence was not impaired in flies with palps surgically removed (as well as those with antennae surgically excised), the discovery of an OR directly stimulated by DEET and other insect repellents and its ORN paves the way for practical applications in repellent R&D. There are a number of applications in reverse chemical ecology for which the “empty neuron system” is an invaluable surrogate. For example, flies carrying appropriate ORs from the malaria mosquito, *An. gambiae* [29] can be used to prospect for novel attractants or repellents, with the benefits of (*i*) not having to deal with a quarantine issues related to maintaining a malaria vector in the lab, and (*ii*) performing single unit recordings on a more amenable insect. Here, the “empty neuron system” is a less desirable alternative. First, Or42a-expressing “empty neuron” does not match the sensitivity of the endogenous ORN sensitive to insect repellents. More importantly, the wild type flies are readily available to laboratories throughout the world, whereas the “empty neuron” still requires, albeit minimal, genetic manipulations. Therefore, we suggest that the ORN in the palpal sensilla pb1 of the fruit fly may be employed as a simple, consistent, and cost-effective tool for screening candidate repellent compounds in the early stages of R&D.

## Materials and Methods

### Olfactory-based choice assay

Tests were performed according to a previously described protocol [14] with slight modifications. In brief, traps (food chambers) were made of 1.5 ml “eppendorf like” micro centrifuge tubes (Sarstedt, NC) and 200 µl pipette tips (USA Scientific, FL). Five microliters of hexane (control) or a hexane solution of a test compound at 100 µg/µl was applied to the stem of a T-shape piece of filter paper (Whatman #1). The stem part of the filter paper was inserted through a slit on the upper part of pipette tip near the entrance of a food chamber so as to preclude flies from walking over the treated surface. Standard *Drosophila* cornmeal diet (UC Davis) was used as food bait. Traps were placed in OPTILUX ™ Petri dishes (100×20 mm style; Becton-Dickinson, NJ) laid with 1% agarose.

#### Single unit recordings

Electrophysiological recordings were performed on 1- to 10-day-old WT 89 and Oregon-R flies. A fly with the proboscis immobilized was mounted on a platform, a glass electrode was placed in the eye and the recording electrode was brought into contact with the base of the sensillum. Stimulation and recording were performed as previously reported for recordings from the fly antennae [19], [33]. DEET, ethyl acetate, ethyl butyrate, ethyl propionate, isoamyl acetate, (*E*)-2-hexenal, heptan-2-one, and 4-methylphenol were purchased from Sigma-Aldrich. IR 3535 and picaridin were gifts from Dr. Kamal Chauhan (USDA-ARS). (+)- and (−)-PMD were purchased from Sigma-Aldrich. Compounds were dissolved in hexane to make stock solutions from which decadic dilutions were made. For stimulus, a 10 µl aliquot of a test compound in the desired dose was loaded on a filter paper strip, the solvent was evaporated for 30 s, and the strip was placed in a disposable plastic syringe from which air was delivered to the preparations. Solvent alone and an empty syringe served as controls. Throughout this article, doses of the stimulus refer to the doses loaded onto stimulus cartridges (source dose). Changes in spike rates during 500 ms stimulation were subtracted from the spontaneous activity of preceding 500 ms, and counts were converted to the conventional scale of spikes/s.

#### Expression of Or42a in the empty neuron system

Test flies (w; Δhalo; UAS-DmOr42a/Or22a-GAL4) were obtained by crossing of transgenic lines (w; CyO/Δhalo; UAS-DmOr42a/TM3 and w; Cyo/Δhalo; Or22a-GAL4) kindly provided by J. R. Carlson (Yale University).
